# Effectiveness of flipped classroom combined with team-, case-, lecture- and evidence-based learning on ophthalmology teaching for eight-year program students

**DOI:** 10.1186/s12909-019-1861-y

**Published:** 2019-11-14

**Authors:** Chun Ding, Shengguo Li, Baihua Chen

**Affiliations:** Department of Ophthalmology, The Second Xiangya Hospital of Central South University, Changsha, 410011 China

**Keywords:** FC-TCLEBL: flipped classroom combined with team-, case-, lecture- and evidence-based learning, LBC: traditional lecture-based classroom, Eight-year program students, Ophthalmology

## Abstract

**Background:**

This study aimed to investigate the benefits and challenges of the flipped classroom combined with team-, case-, lecture- and evidence-based learning (FC-TCLEBL) for ophthalmology teaching for eight-year program students.

**Methods:**

FC-TCLEBL and the traditional lecture-based classroom (LBC) were compared based on student and teacher feedback questionnaires, student learning burden, and scores on standardized tests as well as their effects on the abilities of clinical thinking, scientific research, active-learning, practical application, humanistic care and communication with patients.

**Results:**

Both the students and teachers were more satisfied with the FC-TCLEBL model. More students in the FC-TCLEBL group agreed that the course helped them to develop skills in creative thinking, problem solving, and teamwork. Students in the FC-TCLEBL group spent significantly more time preparing for class than those in the LBC group, but the time spent on review was significantly lower in the FC-TCLEBL group. The students from the FC-TCLEBL group performed better in a post-test on diabetic retinopathy (DR) as compared to the LBC group.

**Conclusions:**

FC-TCLEBL teaching model is effective and suitable for ophthalmology teaching.

## Background

As the development of higher education teaching progresses, methods increasingly emphasize the active participation of students. Moreover, due to the unique qualities of medical education, medical educators have been striving for active-learning abilities, critical thinking skills, good humanistic care, and practical capabilities [1] . Traditional lecture-based classroom (LBC) models cannot meet the needs of modern medical education [2], and thus, a variety of teaching mode reforms have emerged one after another, such as Problem-Based Learning [3], Case-Based Learning [4], Team-Based Learning [5], FC [6], and others. Flipped classroom is a special hybrid teaching [7, 8]. Lage et al. first described the Inverted Classroom Method suitable for Higher Education [9], then Bergman and Sams described FC and used it in school education [10]. This model overturns the traditional order of adding homework elements after classroom teaching [11, 12]. In flipped classroom, students take the initiative to learn before class and solve problems in the process of classroom discussion and cooperation [13] .Different from the traditional passive teaching, flipped classroom is student-centered participatory learning [14–16].

Students complete self-study in their free time. In class, teachers are responsible for guiding students in their communication and answering student questions face-to-face to stimulate student interest in independent learning, promote student participation, and cultivate the students’ innovative thinking and scientific research ability [17]. Since the FC has a great advantage for developing students’ active-learning ability, it is becoming more and more popular among educators and is widely applied in the teaching of medical and nursing courses [18–28]. However, there is no unified method for designing the specific contents of an FC class. In addition, some students in an FC class complained that they spend much more time before class [12, 29–32], have poor absorption and understanding in class, and what they learn is different from what appears in actual clinical cases [9, 21, 33, 34].

TCLEBL is our self-designed and positively responded teaching method for ophthalmology education [9, 21, 33, 34]. Its main aim is to promote student participation and strengthen the students’ understanding of medical knowledge, clinical thinking, and scientific research while effectively reducing the students’ extra-curricular burden. Applying it to an FC model will help to compensate for this mode’s shortcomings, strengthen the effects of teaching, improve the students’ understanding of medical knowledge and clinical reasoning, and optimize their time.

FC-TCLEBL not only improves student interest in learning and their active participation in the process, but it also integrates clinical practice with classroom teaching. In addition, the evidence-based medicine supplemented by teachers greatly improves students’ clinical thinking and scientific research abilities and their ability for reasoning and solving practical clinical problems. It also helps students to get a comprehensive understanding of the disease they are studied. This can all be conducive to the cultivation of medical talent for the twenty-first century and be consistent with the training goals of eight-year clinical medical education.

A large amount of the literature has shown the advantages of the FC model; however, the majority of the work in this area has been performed on undergraduate medical education. It is unclear if these findings can be generalised to graduate medical education. Therefore, further studies aimed at graduate medical education are needed [18]. Our research extended to eight-year medical program students. In addition, our study established a set of rigorous scoring criteria for the assessment of student knowledge of ophthalmological teaching, including for consultation, eye specialist examination, diagnostic decision-making, teamwork, humanistic care, and other clinical skills. The purpose of this study was to provide quantitative data on the effectiveness of the FC-TCLEBL method for communicating ophthalmology knowledge to medical students.

## Methods

### Ethical approval

This study was performed in accordance with the Helsinki Declaration and was approved by the Second Xiangya Hospital Ethics Committee.

### Study design and setting

**Fig. 1:**
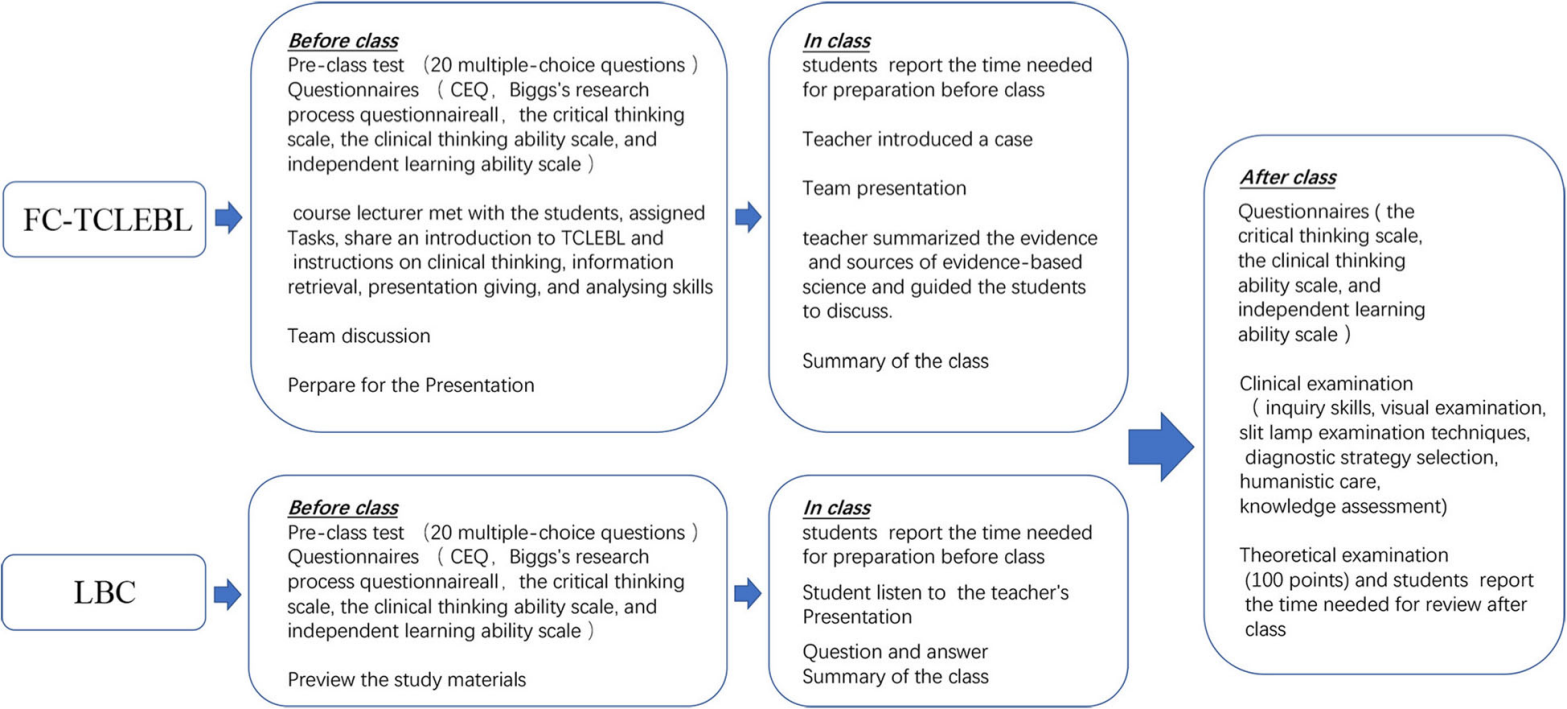
Flowchart illustrating the FC-TCLEBL and LBC models

This study was a comparative study conducted in the medical faculty of Central South University between July 10 and September 31, 2018. A total of 67 eight-year medical program students taking the ophthalmology course were enrolled in the study. The students were allocated into either the FC-TCLEBL group(*n* = 32) or LBC group (*n* = 35). The flowchart of the flipped classroom and traditional lecture-based classroom was summarized in Fig. 1.

We selected DR as a suitable topic for the implementation of the FC-TCLEBL model. We designed an integrated TCLEBL class to engage the students, enhance their understanding of the medical knowledge and clinical rationale, and optimise their study time. A case of DR with decreased visual acuity as the onset symptom was used to explore the process of the TCLEBL model. The objective was to enable the students to obtain a diagnosis, select an appropriate treatment, and apply the learning to analyse and solve clinical problems. Before the classroom session, a course lecturer met with the students to share an introduction to TCLEBL and instructions on clinical thinking, information retrieval, presentation giving, and analysing skills [35]. Previously taught PPTs, teaching videos, and classic cases were sent to the students by QQ or WeChat for studying outside of class. The students needed to search and review the relevant course materials via the Internet and present their questions and difficulties in learning. Teachers and students set up WeChat group, students can ask questions or ask for help from teachers through WeChat, and teachers can provide timely support. To encourage the students to work together and lighten the burden of study, students in the FC-TCLEBL group were further organized into four small teams. Each team was given a topic and prepared a PowerPoint presentation (PPT) for discussion in class. They worked together to prepare the corresponding PPTs, including addressing the questions and difficulties that any of the group members may have had regarding the information on the disease. Each subgroup selected one representative to introduce the clinical manifestations (subgroup 1), anatomy and pathogenesis (subgroup 2), diagnosis and differential diagnosis (subgroup 3), or prevention and treatment (subgroup 4) of the disease. In class, the teacher first introduced a case, each group of representatives make PPT presentation of relevant content. Then the teachers briefly recapped the anatomical and pathological mechanisms of the disease, its clinical manifestations, diagnosis, treatment, and prevention, and specified the key and difficult points in learning about the disease. Finally, the teacher summarized the evidence and sources of evidence-based science and guided the students to discuss.

The LBC group was taught in a traditional classroom manner, and PPTs and other multimedia means were used to explain the medical understanding of DR in class. Both groups were taught by the same teacher, and the teaching contents for the LBC group were consistent with the experimental group. The questions and answers related to the course for the LBC group were also consistent with the experimental group.

### Data collection and analysis

Subjective evaluation: All students (67) and teachers (10) filled in a feedback questionnaire immediately after class. Ramsden’s curriculum experience questionnaire (CEQ) [36] and Biggs’s research process questionnaire [37] were both used in the feedback questionnaire. In addition, all students used the critical thinking scale, the clinical thinking ability scale, and independent learning ability scale for a self-evaluation before and after class. The students were asked to choose the answer that most closely described their actual situation and were told that the questionnaire had nothing to do with the evaluation of their academic performance.

Objective evaluation: A pre-class quiz was performed to assess the students’ baseline understanding of DR knowledge. The quiz contained 20 multiple-choice questions on DR, and each question had the same weight. We calculated the total DR scores for each student. Moreover, the students were required to report the time needed for preparation before class and for review after class. The results for each teaching mode were evaluated by the following clinical and theoretical assessments: 1) Assessment of clinical practice skills (100 points): A specific test after class was set to examine each student’s degree of DR knowledge. Standardized patients (SPs) were used in this assessment. SPs refer to those who after a standardized, systematic training, can consistently and realistically simulate the clinical symptoms of real patients (including with some clinical signs). According to their own impressions, the SPs record and evaluate medical students’ clinical skills and provide teacher-like feedback to students. The evaluation of clinical practice skills mainly included two scores (100 points), a teamwork score (i.e., inquiry skills, visual examination, slit lamp examination techniques, and diagnostic strategy selection; This was tested by putting three students into a group by draw to complete a consultation, visual examination, and slit lamp examination and then to convene and discuss the diagnosis and next treatment plan) and an individual score (humanistic care and knowledge assessment). Each project had a set grading schedule that was graded by the same teacher. 2) Theoretical examination (100 points): At the end of the course, the two groups of students took a final theory examination. The examination questions were set according to the seventh edition of the eighth-year ophthalmology textbook published by the People’s Medical Publishing House. The examination questions, method, and evaluation standard were the same for the two groups. Three teachers made a blind evaluation and gave the theoretical examination scores.

### Statistical analysis

SPSS 22.0 software was used for the statistical analysis. The measurement data are expressed as the mean ± standard deviation. All questionnaire data were analysed using a Mann-Whitney U test. The students’ self-evaluation of their critical thinking, clinical thinking ability, and independent learning ability before and after class were analysed using an paired t-test. The hours spent on class preparation and review were analysed using an independent t-test. The pre-test and post-test scores were also compared between the two groups by an independent t-test. The clinical assessment data were analysed using a chi-square test. A *p* value of less than 0.05 was considered statistically significant.

## Results

A total of 67 eight-year medical program students who were taking the ophthalmology course were enrolled in this study, with 32 students allocated to the FC-TCLEBL group and 35 to the LBC group.

No differences were found between the FC-TCLEBL group and the LBC group in regard to sex (*p* = 1.000) or age (*p* = 0.460). Table 1 shows the demographic data for the two groups.

**Table 1:** Demographic information of medical students who participated in DR study. No differences were found between the FC-TCLEBL group and the LBC group with regard to sex (*p* = 1.000) and age (*p* = 0.460)

		Group				
	All students	FG	TG	Statistics	df	*P* value
Number of students	67	32	35			
Gender						
Male	24	11	13	χ^2^ = 0.056	1	1.000
Female	43	21	22			
Age (years old), mean ± SD	22.896±0.873	22.813±0.859	22.971±0.891	t = 0.742	64.805	0.460

All 67 students submitted the study questionnaires (100%). We compared the students’ views on the FC-TCLEBL and on the LBC versions of the class. Significant differences were found between the FC-TCLEBL group and the LBC group with regard to the teamwork ability questionnaire (*p* = 0.000), analytical skills evaluation scale (*p* = 0.000), and problem-solving skills evaluation scale (*p* = 0.002). Table 2 shows the data for the two groups.

**Table 2:** The questionnaire was answered by all the students after the examination. The anonymous paper questionnaire survey was adopted to compare the subjective learning feelings of the two groups of students. The content includes skills development, appropriate assessment, and academic environment. 1 = strongly agree, 2 = agree, 3 = neutral, 4 = strongly disagree, 5 = disagree. Effect size is calculated by test statistic divided by the root of sample size (small effect: 0.1 < r ≤ 0.3, medium effect: 0.3 < r ≤ 0.5, large effect: r > 0.5)

	Flipped classroom	Control	Mann-Whitney U	*P* value	Effect size
1. Skills Development					
The class has helped me develop my ability to work as a team member.	1.6875 ± 0.89577863	3.514285714± 0.939435832	102.000	0.000	0.723519
The class has sharpened my analytical skills.	2.03 ± 0.97	3.11±0.97	249.500	0.000	0.493219
As a result of my degree course, I feel confident about tackling unfamiliar problems.	2.531 ± 1.27	3.143± 0.961	393.500	0.031	0.263531
The class has developed my problem-solving skills	2.4063 ± 0.9456	3.1429± 0.971	331.500	0.002	0.378375
The class has improved my skills in written communication	2.65625 ± 1.09572115	3.342857143±0.903256894	339.500	0.003	0.366768
My class has helped me to develop the ability to plan my own work	2.53125 ±1.163542283	3.085714286±1.158817131	389.500	0.025	0.273183
2. Appropriate Assessment					
There is a lot of pressure on me as a student in this class	2.9687± 1.092034946	3.257142857±1.125110224	483.500	0.314	0.12303
The workload is too heavy	2.9375±1.014014697	3.085714286±1.267418323	502.500	0.423	0.097862
I am generally given enough time to understand the things I have to learn	2.4375±1.075759297	3.085714286±1.401529776	359.000	0.008	0.32413
The sheer volume of work to be got through in this class means it can’t all be thoroughly comprehended Clear Goals and Standards	2.75±1.163975	2.8±1.656157	539.500	0.788	0.032865
I have usually had a clear idea of where I am going and what is expected of me in this class	2.0625±1.075759297	2.628571429±1.884565547	418.000	0.065	0.225412
It is always easy to know the standard of work expected	2.13±1.24	2.94±1.92	345.000	0.006	0.339157
The staff made it clear right from the start what they expected from students	2.3438±0.9708	3.1429±2.0616	345.00	0.005	0.340501
It has often been hard to discover what is expected of me in this class	4.125±0.907	3.3429±2.1667	357.500	0.008	0.322663
3. Academic Environment					
The class is intellectually stimulating.	2.0625±1.075759297	2.742857143±2.418677324	392.000	0.030	0.265852
The class administration is effective in supporting my learning	2±1.135924	3.114286±2.443488	268.000	0.000	0.46292
My class has stimulated my enthusiasm for further learning	2.1875±1.148281	3±2.621644	340.000	0.004	0.348076
Where it was used, information technology helped me to learn	2.28125±1.32554	2.942857±2.695528	368.500	0.013	0.303115
I feel part of a group of students and staff committed to learning	2.46875±1.243937	2.971429±2.882211	408.50	0.048	0.241662
I feel I benefit from being in contact with active researchers	2.25±1.04727	3±3.075118	359.500	0.009	0.31741

Tables 3 summarizes the feedback from the teachers who participated in the FC-TCLEBL and LBC classes. As compared to the LBC class, more teachers believed that the FC-TCLEBL model enhanced student understanding of the topic (*p* = 0.001). Furthermore, the teachers enjoyed the methods used in the FC-TCLEBL class (*p* = 0.029). In addition, the teachers agreed that the FC-TCLEBL model met their expectations (*p* = 0.045).

**Table 3:** Comparison of teachers’ perspectives between the FC-TCLEBL group and the LBC group. 1 = strongly agree, 2 = agree, 3 = neutral, 4 = strongly disagree, 5 = disagree. Effect size is calculated by test statistic divided by the root of sample size (small effect: 0.1 < r ≤ 0.3, medium effect: 0.3 < r ≤ 0.5, large effect: r > 0.5)

	Flipped classroom	Control	Mann-Whitney U	*P* value	Effect size
The lecture greatly enhances students’ understanding about this topic.	1.5±0.527046	2.9±0.737864787	7.500	0.001	0.41234
The class met my expectations.	2.3±0.823273	3.1±0.737865	25.500	0.045	0.244349
It is an enjoyable way of teaching.	1.9±0.737865	2.8±0.918936583	22.500	0.029	0.266585
Overall, I am satisfied with the quality of this class.	2.6±0.966092	3.7±1.159502	24.000	0.043	0.247648
The climate of this class is conducive to learning for students.	2.2±1.032796	3.7±0.948683	15.000	0.007	0.332071

**Fig. 2:**
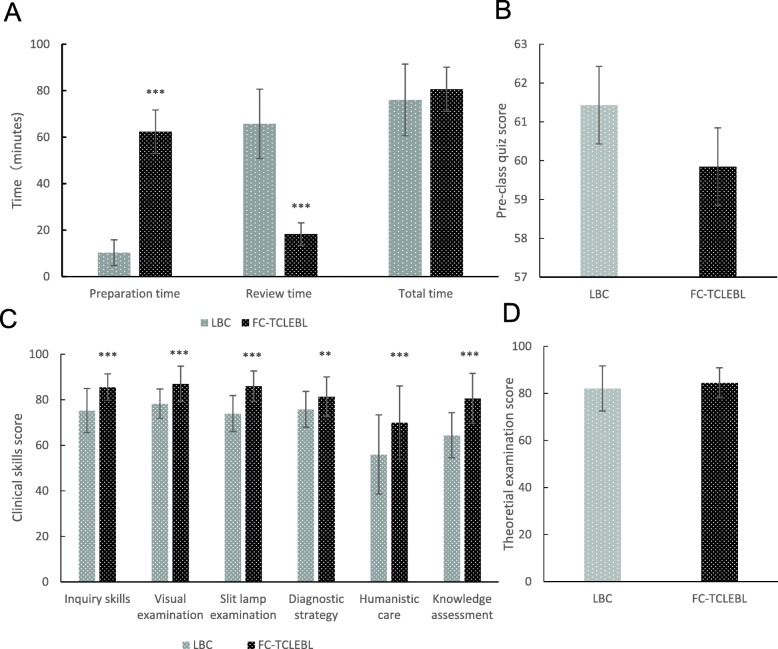
Comparison of students’ feedback between the FC-TCLEBL group and the LBC group. **a** The students in the FC-TCLEBL group spent significantly more time preparing for class, but spent less time on review. There was no significant difference in total time consumption between the two groups. **b** Two groups students took a pre-class quiz on DR. There were no statistical differences between the two groups. **c** The scores of inquiry skills, visual examination, slit lamp examination techniques, diagnostic strategy, humanistic care and knowledge assessment in the FC-TCLEBL group were higher than that in the LBC group. **d** The difference was not statistically significant in the theoretical test score between the FC-TCLEBL group and the LBC group. * indicates 0.05 > *p* > 0.01, ** indicates 0.01 > *p* > 0.001, *** indicates *p* < 0.0001. For details of statistical analyses of data presented in all figures, see results section

Figure 2 compares the time spent for the FC-TCLEBL and LBC classes. The students in the FC-TCLEBL group spent significantly more time preparing for class than those in the lecture-based classroom group (62.344 ± 9.331 min vs. 10.286 ± 5.550 min, *p* < 0.001). The time spent on review was significantly lower in the FC-TCLEBL group (18.281 ± 4.854 min vs. 65.714 ± 14.909 min, *p* < 0.001). No significant difference was found for the total time between the two groups (80.625 ± 9.483 min vs. 76 ± 15.425 min, *p* = 0.141).

The two groups of students took a pre-class quiz on DR. No statistical differences were found between the two groups (59.844 ± 12.014 vs. 61.429 ± 14.275, *p* = 0.626). Figure 2 presents the results.

The results of the clinical practice skills assessment of the two groups were also compared. The teamwork scores for inquiry skills (85.563 ± 5.808 vs. 75.257 ± 9.727, *p* = 0.000), visual examination (87 ± 7.721 vs. 78.286 ± 6.438, *p* = 0.000), slit lamp examination techniques (86 ± 6.643 vs. 73.943 ± 7.874, p = 0.000), and diagnostic strategy selection (81.375 ± 8.657 vs. 75.829 ± 7.857, *p* = 0.008) were higher in the FC-TCLEBL group than in the traditional lecture-based classroom group. The individual aspects of humanistic care (70 ± 16.064 vs. 56 ± 17.354, *p* = 0.001) and knowledge assessment (80.625 ± 10.980 vs. 64.429 ± 9.909, p = 0.000) were compared between the two groups, and the difference was statistically significant. The results are shown in Fig. 2.

The theoretical average test score of the students in the flipped classroom group was 84.5 ± 6.370 and in the traditional lecture-based classroom group was 82.1 ± 9.585. The difference was not statistically significant (t = − 1.216, *p* = 0.229). The results are shown in Fig. 2.

**Fig. 3:**
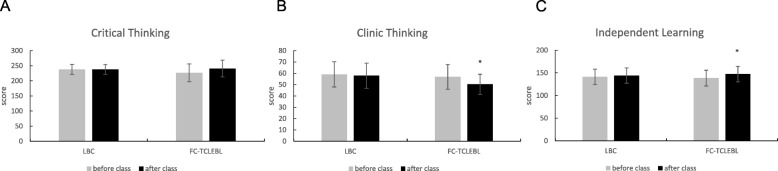
Comparison of the self-evaluation of students’ critical thinking, clinical thinking ability and independent learning ability before and after class. **a** There was no significant difference in self-evaluation of students’ critical thinking before and after class in both FC-TCLEBL group and LBC group. **b** The student in the FC-TCLEBL group have improved their clinical thinking ability (56.84375 ± 10.98124 vs 50.375 ± 8.870938, *p* = 0.01188) after class. **c** The student in the FC-TCLEBL group have improved independent learning ability (138.5 ± 17.40967 vs 147.3125 ± 17.32132, *p* = 0.046666) after class

We compared the students’ self-evaluation of their critical thinking, clinical thinking ability, and independent learning ability before and after class. The students in the FC-TCLEBL group improved their clinical thinking ability (56.84375 ± 10.98124 vs. 50.375 ± 8.870938, *p* = 0.01188) and independent learning ability (138.5 ± 17.40967 vs. 147.3125 ± 17.32132, *p* = 0.046666) after class. However, the students in the LBC group had no improvement in critical thinking, clinical thinking ability, or independent learning ability after class. The results are shown in Fig. 3.

## Discussion

The team-based learning in the FC-TCLEBL model was a very effective method for learning. First, all members were required to complete part of the study independently, which may have been able to improve the students’ independent learning ability. Therefore, the students in FC-TCLEBL group believed that their independent learning ability had improved after the class. Second, for FC-TCLEBL group, although the preparation time was significantly longer, the review time after class was significantly shortened compared with LBC group. There are no significantly difference on total time between FC-TCLEBL group and LBC group. So as compared to LBC group, the students in the FC-TCLEBL group didn’t complain that the workload was heavy. Third, not only could every student actively join in the learning activities, express their own views and ideas, and intensify exchange and communication, but they could also cultivate their awareness of intra-group cooperation and inter-group competition, enjoying teamwork while learning cooperatively. Thus, it could also cultivate the students’ humanistic care and teamwork abilities, so students in FC-TCLEBL group received higher scores for teamwork and humanistic care.

Case-based learning in the FC-TCLEBL model is characterized by its vividness, objectivity, and authenticity, and it analyses typical cases in detail, which are easy for students to understand and master. Case-based learning teaches students to solve problems by carefully selecting typical cases in combination with clinical practice in order to give students an experience of clinical practice, let them realize the relationship between theory and practice, help them step into practice from theory, establish problem-solving awareness, and cultivate a good way of thinking. Therefore, when the two groups of students used the clinical thinking ability scale for self-evaluation before and after class, the students in the FC-TCLEBL group reported that their clinical thinking ability was significantly improved after class.

All students completed a pre-class quiz. In the pre-class quiz, no statistical differences were found between the FC-TCLEBL group and the LBC group. Thus, no difference was observed in the basic knowledge on DR between the two groups, which is consistent with the results in Lin [12]. Furthermore, we gave the students a post-class exam that established a rigorous set of scoring criteria to assess their acquisition of knowledge on DR and their ability to apply their learning, including for consultation, eye specialist examination, diagnostic decision making, teamwork, humanistic care, and other clinical skills. The post-class exam score showed significant differences between the two groups. The FC-TCLEBL teaching method was beneficial for improving the students’ clinical operational ability. The results of this study showed that the scores of the FC-TCLEBL group were higher than that of the LBC group in the assessment of clinical practice skills, especially in the three aspects of diagnostic strategy, humanistic care, and overall evaluation (overall clinical competence). As compared with the LBC mode, the FC-TCLEBL approach could improve the students’ clinical comprehensive practice ability, train students in the correct clinical work, strengthen comprehensive and systematic clinical thinking ability, improve the students’ ability to analyse and solve problems, and improve the professional quality of the medical students and their humanistic qualities. In the examination of clinical thinking and clinical operational skills, the aspects of consultation, operational skills, diagnostic decision making, and basic knowledge assessment were on DR, therefore, the students undergoing the FC-TCLEBL teaching approach on DR had better performance. In addition, in the examination of clinical thinking and clinical operational skills, the team scores were used to examine the students’ teamwork ability. As the students of the FC-TCLEBL group already had a basis for teamwork and expressed positive feelings about the effectiveness of team-based learning [38], their team scores were high. Overall, the students in the FC-TCLEBL group obtained higher scores. The other explanations for this result include the following: 1) The study materials provided before class (including the video and manuscript materials) were specific and detailed, covering most of the examination content. The classroom discussion questions were set according to the examination focus, and this condition made it more conducive for students to grasp the examination focus. 2) The pre-class preparation and in-class presentations and discussions highlighted the leading position of the students in the teaching process, provided students with the opportunity to exhibit independent thinking, amplified the enthusiasm for learning, and promoted the students’ mastery of the knowledge on DR.

Without decreasing the basic knowledge, the FC approach strengthens the interactivity among students in the classroom, highlights the leading position of the students in the teaching process, provides students with the opportunity to display independent thinking, and improves the enthusiasm for learning. This increase in interest on both emotional and cognitive levels is beneficial for learning [39]. The teacher is no longer at the centre of the classroom. Students can ask questions about what they do not understand, express their own understanding of a problem, and understand the other students’ thoughts on a problem, which are all conducive to the learning of a basic operating knowledge and clinical case analysis. So, both teachers and students were more satisfied with the FC-TCLEBL class, according to the feedback questionnaire given after class. Previous studies have shown that the FC helps to cultivate students’ innovation ability, collaboration ability, and collective cohesion [12, 29, 40]. Our findings were consistent with previous studies that showed that the FC approach improves student performance [19, 21, 28]. Therefore, we believe that FC-TCLEBL teaching for DR is more appropriate than an LBC model. The FC-TCLEBL approach did not increase the scores of the students on the final exam, which is consistent with the results of Lin [12].

### Limitations

There are some limitations to our research that are worth considering. First, our research on the FC-TCLEBL model was limited to a single chapter on DR, and it did not involve how to reasonably choose the themes or frequency for using the FC-TCLEBL model. Second, although we have achieved good results in the implementation of the FC-TCLEBL model with 67 eight-year postgraduates, the sample size was relatively small, and the next step for this research will be to extend it to the clinical teaching of a larger group of postgraduates majoring in ophthalmology. Third, our study conducted pre-class and post-class tests to evaluate the learning effect; however, there was a lack of scoring of student learning performance in the course. Further study will include course performance scoring to evaluate the teaching effect of the FC-TCLEBL model more comprehensively and accurately. Fourth, in our study, we did not compare the effects of FC, TBL and CBL respectively. Further research is needed to compare the functions of these three teaching modes. In addition, due to time constraints, we did not require students to write scientific research papers, and we lack specific indicators to evaluate the students’ scientific research abilities. However, we will further track the students’ future performance and paper publications.

## Conclusion

Taken together, the students and teachers approved of the FC-TCLEBL teaching mode. Although the pre-class preparation time increased, the students did not feel that the preparation workload was too heavy, and the post-class review time was significantly shortened. The performance of the students of the FC-TCLEBL group improved significantly in the clinical knowledge examination for DR, and the students’ knowledge acquisition ability and application were also enhanced. As the FC-TCLEBL teaching on DR was only a one-time occurrence, no significant difference was found in the final theoretical test scores of the two groups. Future research should increase the sample size to optimize the theme and frequency of the FC in ophthalmology teaching.

## Data Availability

The datasets used and/or analysed during the current study are available from the corresponding author on reasonable request.
